# Morphological Characteristics of Terminalia of the Wasp-Mimicking Fly, *Stomorhina discolor* (Fabricius)

**DOI:** 10.3390/insects8010011

**Published:** 2017-01-12

**Authors:** Kittikhun Moophayak, Sangob Sanit, Tarinee Chaiwong, Kom Sukontason, Hiromu Kurahashi, Kabkaew L. Sukontason, Roy C. Vogtsberger, Nophawan Bunchu

**Affiliations:** 1 Nakhonsawan Campus, Mahidol University, 402/1 Moo 5, Khaothong Subdistrict, Phayuhakiri District, Nakhonsawan 60130, Thailand; khun_khithop@hotmail.com; 2 Department of Parasitology, Faculty of Medicine, Chiang Mai University, 110 Inthavaroros Road, Muang District, Chiang Mai 50200, Thailand; sang.ngeab@gmail.com (S.S.); kom.s@cmu.ac.th (K.S.); kabkaew.s@cmu.ac.th (K.L.S.); 3 College of Medicine and Public Health, Ubon Ratchathani University, 85 Sathonlamark Road, Warin Chamrap District, Ubon Ratchathani 34190, Thailand; trainee.4004@gmail.com; 4 Department of Medical Entomology, National Institute of Infectious Diseases, Tokyo 162-8640, Japan; mlb15110@nifty.com; 5 Department of Biology, Midwestern State University, 3410 Taft Boulevard, Wichita Falls, TX 76308, USA; roy.vogtsberger@mwsu.edu; 6 Department of Microbiology and Parasitology, Faculty of Medical Science, Naresuan University, 99 Moo 9, Tha Pho Subdistrict, Muang District, Phitsanulok 65000, Thailand; 7 Centre of Excellence in Medical Biotechnology, Faculty of Medical Science, Naresuan University, 99 Moo 9, Tha Pho Subdistrict, Muang District, Phitsanulok 65000, Thailand

**Keywords:** *Stomorhina discolor*, ultrastructure, external genitalia, ovipositors

## Abstract

*Stomorhina discolor* (Fabricius), a species of blow fly that mimics wasps, is distributed worldwide, but detailed information about characteristics of its adult terminalia is incomplete. To help fill this gap in the information, the morphology of adult stages of *S. discolor* was investigated using light microscopy and scanning electron microscopy. Observations using the light microscope revealed unique characteristics of the male genitalia that are markedly different from other blow fly species. More morphological detail, including observation of several sensilla (e.g., sensilla trichoid and sensilla basiconica) along the male terminalia and female ovipositor, was seen under the scanning electron microscope. These details can be taxonomically valuable for identifying males and females of *S. discolor* and may help address matters concerning copulation in this species.

## 1. Introduction

*Stomorhina discolor* (Fabricius, 1794) is a blow fly species in the subfamily Rhiniinae and is known as the wasp-mimicking fly. It has been recorded in many parts of the world [1,2,3,4,5]. Biological information for this species is limited. In Thailand, research in Phitsanulok Province of the lower northern region of the country revealed that this species was the most commonly collected blow fly species of the subfamily Rhiniinae, and it can be found in several types of areas including agricultural, mountainous, and forested areas [6]. Senior-White et al. [4] noted that larvae of this species were found in nests of the ant *Camponotus angusticollis* (Jerdon), among the roots of *Hevea brasiliensis* Muell. Arg. affected by the fungus *Botryodiplodia theobromae* Patton, and riddled with small boring beetles. Adults are occasionally attracted by opening an ant nest. Males are known to often exhibit a hovering behavior in small swarms under trees [4]. Among the morphological descriptions of *S. discolor* that have been published, only limited terminalia illustrations of male genitalia of the species have been provided [4]. Molecular phylogeny of this species has been analyzed by Kutty et al. [7].

In order to elaborate the biological information of this fly species, morphological features of both male and female terminalia of *S. discolor* were studied by using light microscopy (LM) and scanning electron microscopy (SEM).

## 2. Materials and Methods

### 2.1. Wasp-Mimicking Fly, S. discolor

In December 2012, we discovered numerous blow fly specimens of *S. discolor* near a blooming *Buchanania lanzan* Spreng tree at Mahidol University Nakhonsawan Campus, Nakhonsawan Province in the north-central part of Thailand (15.58189° N, 100.14640° E). Individual flies were observed moving over the flowers and were collected using a sweep net. They were anesthetized in bottles with ethyl acetate solution and transported back to the lab for species confirmation under a stereomicroscope (Olympus, Tokyo, Japan) using the taxonomic key of Kurahashi and Bunchu [2].

**Figure 1:**
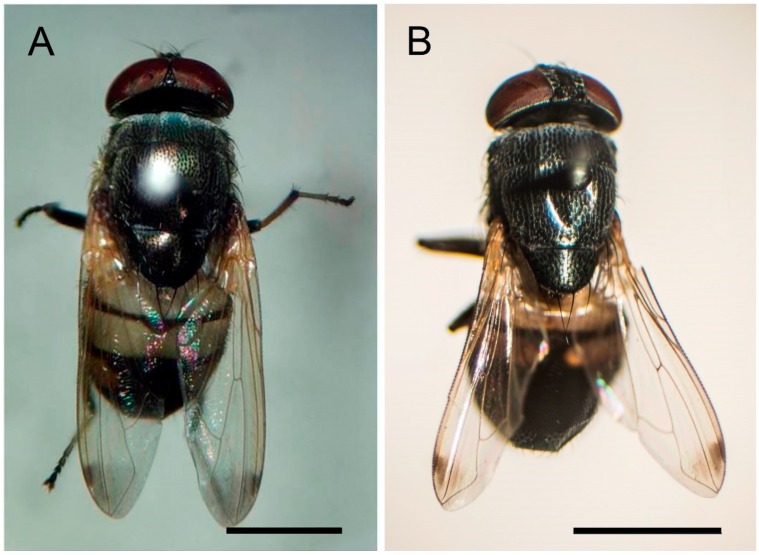
Dorsal view of *Stomorhina discolor*. (**A**) Male with holoptic eyes; (**B**) Female with dichoptic eyes (bar = 2 mm).

As seen in Figure 1, members of *S. discolor* are small-medium size blow flies with a dark colored thorax with dense pollinosity. Wings are transparent with an apical dark spot in both sexes. The abdomen is black or brown posteriorly with a contrasting yellow on tergites 1 + 2. Males are easily distinguished from females by their holoptic eyes; whereas, the eyes of females are dichoptic. After specific identity of specimens had been confirmed, male and female flies of *S. discolor* were individually selected to further investigate the ultrastructural morphology of their external genitalia.

### 2.2. Light Microscopy

Fifteen fly specimens of each sex were cut between tergites 4 and 5 using a sharp forceps under a stereomicroscope to separate the terminalia in order to eventually manipulate the genitalia at optimal observational angles. The terminalia were then individually macerated in a mixture of 2 mL of 10% (w/v) potassium hydroxide (KOH) solution and 0.12 mL of 95% ethanol in each well of a 12-well plate for 4 d. Specimens were rinsed once with distilled water to remove the caustic KOH solution and transferred into a 0.85% sodium chloride (NaCl) solution on a dissecting plate so that the genitalia could be dissected from the terminalia. Male genitalia and female ovipositors were gently separated from the macerated tissue using fine needles (No. 23) and sharp forceps under the stereomicroscope (Olympus). Each specimen was photographed using a digital camera (Samsung S700; Samsung, Seoul, Korea) and illustrated by painting with Chinese ink.

### 2.3. Scanning Electron Microscopy

Three other specimens of each sex were examined by scanning electron microscopy. All specimens were dissected as previously described in the light microscopic portion of this study. The terminalia were cleaned by soaking in phosphate buffer (PB, pH 7.4) to remove contamination artifacts. After that, they were primary fixed in 2.5% glutaraldehyde at 4 °C for 24 h. They were then rinsed with PB three times at 15 min intervals and post-fixed with 1% osmium tetroxide at room temperature for 1 h. Postfixation was followed by rinsing again as previously described and dehydrating with increasing alcohol concentrations as follow: 70%, 80%, 90%, and 95%. Specimens were left in each alcohol concentration for 12 h. After the gradual dehydration process in the alcohol series, the specimens were dehydrated with absolute alcohol for two 12 h periods. Finally, they were subjected to critical point drying, attached to aluminum stubs with carbon double-stick tape, and coated with gold in a sputter-coating apparatus. The gold–coated specimens were then examined under a JEOL JSM-6610LV scanning electron microscope (JEOL, Peabody, MA, USA) at the Medical Science Research Equipment Center, Faculty of Medicine, Chiang Mai University in Chiang Mai, Thailand. The terminology used for most fly structures in this study follows the descriptions of Bernhard and Haenni [8], but the sensilla terminology specifically follows that of Zacharuk [9].

## 3. Results

**Figure 2:**
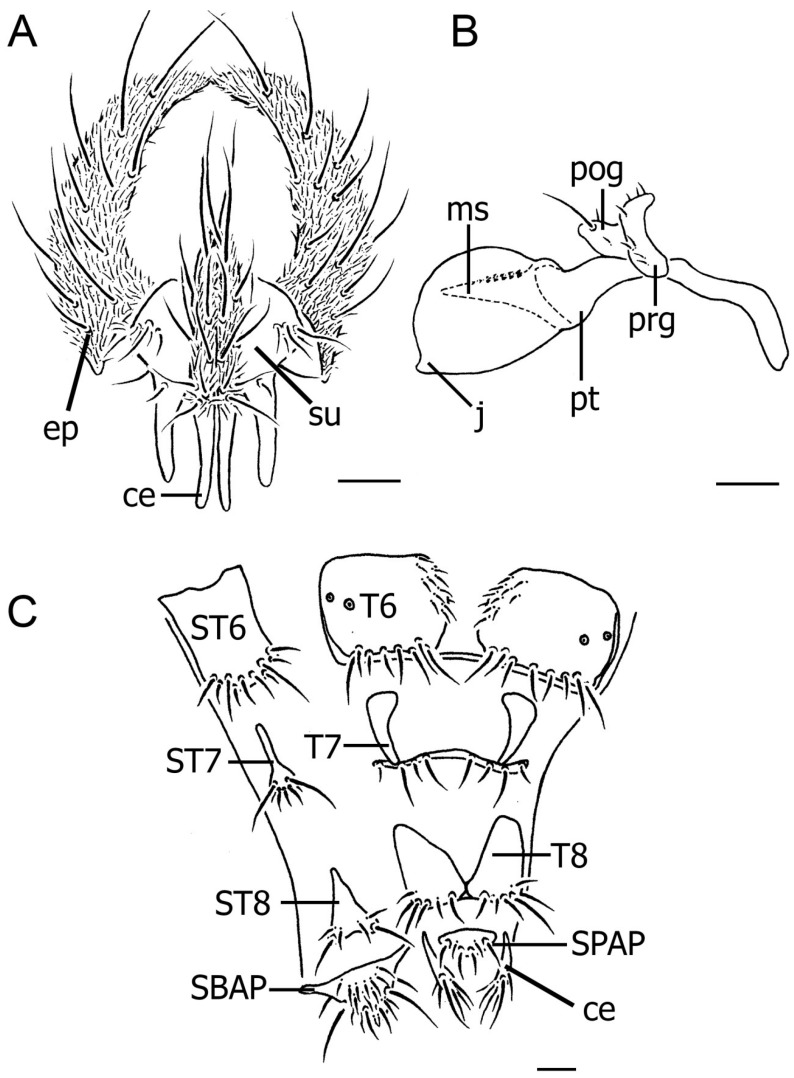
Illustrations of genitalia of *Stomorhina discolor*. (**A**) Posterior view of external male genitalia demonstrating prominent features: cercus (ce), surstylus (su), and epandrium (ep). Paired longitudinal cerci (ce) lie medial to the shorter surstyli (su) (bar = 2 mm); (**B**) Lateral view of aedeagus showing pregonite (prg), postgonite (pog), phallic tube (pt), median stylus (ms), and juxta (j) (bar = 2 mm); (**C**) Ovipositor showing tergite 6 (T6), sternite 6 (ST6), tergite 7 (T7), sternite 7 (ST7), tergite 8 (T8), sternite 8 (ST8), cercus (ce), subanal plate (SBAP), and supra-anal plate (SPAP) (bar = 0.1 mm).

A total of 36 specimens of *S. discolor* were examined by light and scanning electron microscopy. The average body length (head–abdomen) of all specimens was 6.88 mm, varying from 4.80–9.30 mm, but the distinct features were similar. Characteristic features of the terminalia of *S. discolor* are softly sclerized and formed posterior to the fifth abdominal segment as external reproductive organs. Examination under light microscopy showed that male terminalia were hardened and consisted of epandria, surstyli, and cerci (Figure 2A), whereas the tube-like ovipositor of the female consisted of the slightly sclerotized plates of sternites and tergites 6–8, the supra-anal plate (SPAP), and the subanal plate (SBAP) coupled with the cerci (Figure 2C). Various kinds of microtrichia are present on the surface of the genitalia of both sexes. The extended aedeagus is illustrated in Figure 2B.

**Figure 3:**
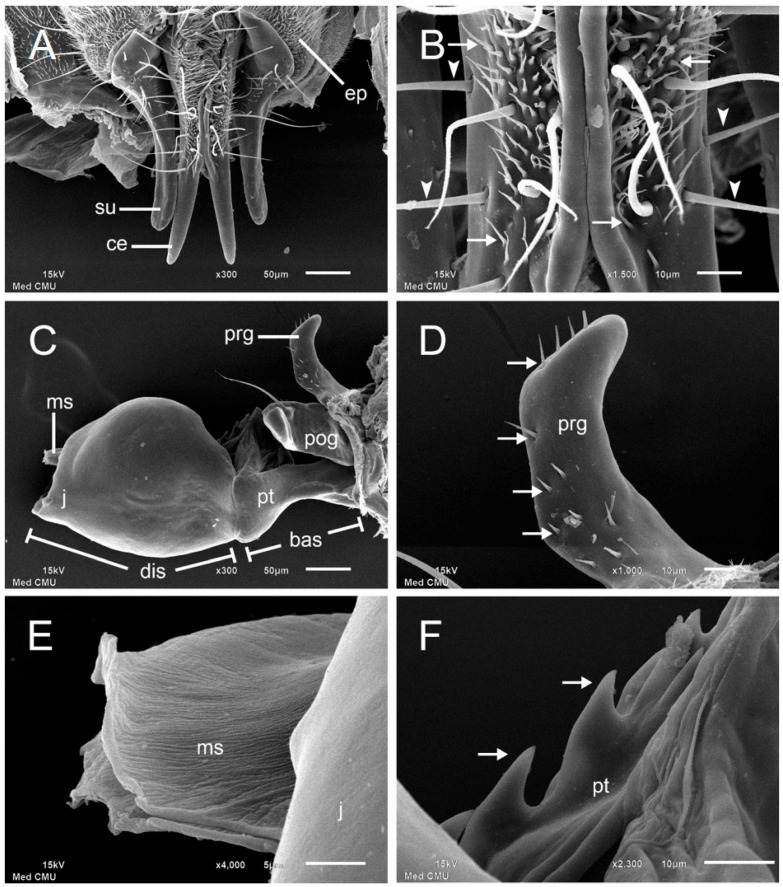
SEM micrographs of external male genitalia of *Stomorhina discolor*. (**A**) Posterior view of genitalia demonstrating prominent features: cercus (ce), surstylus (su), and epandrium (ep). Paired longitudinal cerci (ce) lie medial to the shorter surstyli (su); (**B**) High magnification of proximal portion of cerci showing sensilla trichodea of variable lengths (arrowheads) and sensilla basiconica with short hair shafts (arrows); (**C**) Lateral view of aedeagus showing pregonite (prg), postgonite (pog), phallic tube (pt), basiphallus (bas), distiphallus (dis), juxta (j), and median stylus (ms). The median stylus (ms) is inserted within the apically-oriented juxta (j). The pregonite (prg) and postgonite (pog) bearing a single bristle are prominent projections at the base; (**D**) High magnification of pregonite (prg) revealing sensilla basiconica with short hair shafts (arrows); (**E**) High magnification of median stylus (ms) with rough surface and expanded apex; (**F**) High magnification of phallic tube with dorsal serration (arrows) near center of aedeagus.

The dorsal view of the male genitalia under SEM revealed prominent features of the cercus, surstylus, and epandrium (Figure 3A). The cerci are slender and diverge distally with tapering tips (Figure 3A). The medial edges of the cerci are joined toward the base and are raised dorsally above the surface as a ridge (Figure 3A,B). The integument of the cercus is mostly covered in the proximal half with sensilla basiconica with short hair shafts and sparsely covered with sensilla trichodea (Figure 3B). The surstylus is basally enlarged in the proximal half and gradually tapered posteriorly in the distal half (Figure 3A). Strong bristles are only located in the proximal half of the surstylus. The medially located cerci are significantly longer and more slender than the surstyli that flank them laterally (Figure 3A). The epandria are densely covered with microtrichia and strong bristles (Figure 3A). SEM micrographs of the internal male genitalia or aedeagus revealed some more prominent components of the organ consisting of the pregonite, postgonite, phallic tube, juxta, and median stylus (Figure 3C). Surfaces of the postgonite, phallic tube, and juxta are all smooth, with the exception of a single, strong preapical bristle on the postgonite (Figure 3C). The pregonite bears numerous sensilla basiconica with short hair shafts as seen in Figure 3D. Within the juxta at the base of the aedeagus, the membranous tubular stylus is roughened on the surface and expanded apically (Figure 3E). Near the center of the extended aedeagus, a serrated median, dorsal process was presented (Figure 3F).

**Figure 4:**
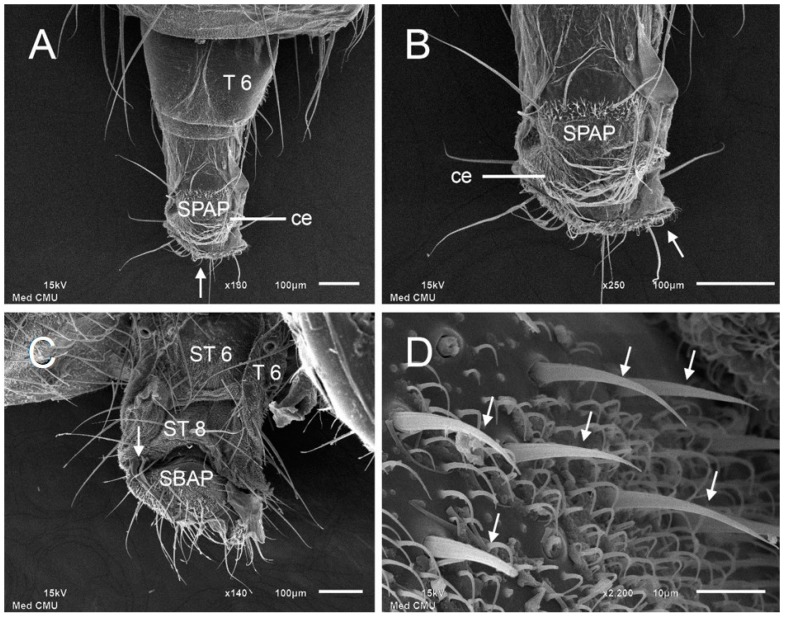
SEM micrographs of ovipositor of *Stomorhina discolor*. (**A**) Dorsal view of ovipositor showing tergite 6 (T6), supra-anal plate (SPAP), and cercus (ce). Gonopore at apex of ovipositor (arrow); (**B**) High magnification of dorsal view of ovipositor showing more detail of supra-anal plate (SPAP) and cercus (ce). Gonopore (arrow); (**C**) Ventral view of ovipositor showing side of tergite 6 (T6), sternite 6 (ST6), sternite 8 (ST8), and subanal plate (SBAP). Gonopore (arrow); (**D**) High magnification of subanal plate (SBAP) showing sensilla basiconica (arrows) and smaller microtrichia.

The ovipositor of *S. discolor* is a flexible tubular structure which is usually withdrawn and concealed inside the abdomen. It extends outward from the fifth abdominal segment when depositing eggs. This structure is composed of more sclerotized plates consisting of paired tergites 6–8, sternites 6–8, paired cerci, the supra-anal plate (SPAP), and the subanal plate (SBAP), all joined together by a softer, less sclerotized membranous exoskeleton (Figure 2C). A dorsal view of the ovipositor under SEM showed that the sixth tergites are relatively smooth sclerotized plates with only a group of hairs at their apices (Figure 4A). In the ventral view under SEM (Figure 4C), sternites 6–8 are seen to be covered with numerous short hairs and a few long bristles near the apex of each. Sensilla trichoid are widespread on the external ovipositor and subanal plate (SBAP) (Figure 4C). The gonopore is located distally in the ovipositor (Figure 4B,C). Higher magnification provided by SEM reveals sensilla basiconica on the subanal plate (SBAP) as is pointed out in Figure 4D.

## 4. Discussion

A more complete description of the external genitalia of both sexes of *S. discolor* is being reported for the first time in this study. Distinct characteristics of the terminalia of *S. discolor* are unique to the species and are significantly different from those of other blow fly species. Illustrations of select male genitalia of some species of the genus *Stomorhina* of the British India fauna (including *S. discolor*) have been previously published [4]. Results of the current study have some similarity to the study of Senior-White et al. [4]; however, more specific details of each component of the genitalia are demonstrated in this study. Rognes [10] provided illustrations of the male and female genitalia of *Stomorhina lunata* (Fabricius) which are distinctly different from *S. discolor* (Table 1). Two types of sensilla, including sensilla trichodea and sensilla basiconica, were found on both the male and female genitalia of *S*. *discolor* in the present study. Likewise, the ovipositor of *Ceylonomyia nigripes* (Aubertin) is equipped with both sensilla trichodea and sensilla basiconica, in addition to sensilla placodea and sensilla styloconica [11]. Sensilla on terminalia are used for the positioning of genitalia for copulation during mating events [12]. Wang et al. [13] examined the expression pattern of an odorant receptor in the ovipositor of the blow fly *Lucilia sericata* (Meigen). Therefore, sensilla on the ovipositor may also have an olfactory function. In addition, sensilla on the female terminalia of the mosquito *Aedes aegypti* (Linnaeus) have been proposed to play a role in copulation as well as in ovipositional behavior [14]. However, sensilla of identical structures possibly serve different functions in different species of fruit fly, *Drosophila* spp. [12].

## 5. Conclusions

In conclusion, this study provides several distinctive details of the external terminalia of *S*. *discolor* which are not only useful for taxonomic purposes, but also provide important revelations that may be useful in future functional studies of the external genitalia of this fly species.

## Figures and Tables

**Table 1:** Comparison male and female terminalia of *Stomorhina discolor* (current study) and *Stomorhina lunata* [10].

Characteristics	*Stomorhina discolor*	*Stomorhina lunata*
Body length	4.80–9.30 mm	5.00–9.00 mm
Male genitalia		
Cercus	slender with tapering tips	with cleft distal half
Surstylus	enlarged in proximal half tapered in distal half	narrow and pointed in lateral view
Pregonite	with short hair shafts at tip and posterior	with few setae posteriorly
Postgonite	with a strong preapical bristle	with a strong basal seta
Phallic tube	with dorsal serration	undetermined
Median stylus	with rough surface expanded at apex	undetermined
Female ovipositors		
Tergite 7	with broad sclerotize in between 2 half separates	with narrow sclerotize in between two half separates
Sternite 7	rod-like with expanded at apex	square-like
Sternite 8	narrow triangular	square-like with concave at apex
Subanal plate	broad triangular with expanded at base	broad triangular
Supra-anal plate	short and narrow triangular	short and broad triangular
